# Laboratory to Clinical Investigation of Carbapenem Resistant *Acinetobacter baumannii *Outbreak in a General Hospital

**DOI:** 10.5812/jjm.13120

**Published:** 2014-01-01

**Authors:** Mo Guo-xin, She Dan-yang, Guan Xi-zhou, Cui Jun-chang, Wang Rui, Cui Zhi-gang, Chen Liang-an

**Affiliations:** 1 Department of Respiratory Disease, Chinese PLA General Hospital, Beijing, China; 2 Institutes of Clinical Pharmacology Chinese, PLA General Hospital, Beijing, China; 3 State Key Laboratory for Communicable Diseases, Prevention and Control Institute of Communicable Disease Prevention and Control Chinese Center for Disease Control and Prevention, Beijing, China

**Keywords:** *Acinetobacter baumannii*, Drug Resistanc, Sequence Homology, Cross Infection

## Abstract

**Background:**

The number of reported cases, infected with carbepenem resistant *Acinetobacter baumannii* (CRAb) and multi-drug resistant (MDR) *Acinetobacter *species had gradually increased in most PLA general hospital wards from April to June in 2007.

**Objectives:**

We have described the investigation of an outbreak of CRAb and MDR *Acinetobacter* in PLA general hospital, Beijing. The prospective and retrospective findings were identified and analyzed to study the infection causes.

**Materials and Methods:**

*A. baumannii* samples were collected from the patients and environment in each hospital unit. The onset times were recorded according to their case information. All samples were characterized by genotype and compared using pulsed-field gel electrophoresis (PFGE). The microorganism susceptibility was tested using the in vitro minimal inhibitory concentration (MIC) breakpoints method.

**Results:**

A total of 69 *A. baumannii* strains were successfully isolated from 53 patients. About 89.1% of them were resistant to ampicillin and 89.2% to cefotaxime and 75.4% to all standard antibiotics. PFGE analysis revealed that nine of the isolates had unique clones and the epidemic clone types were A, B and C.

**Conclusions:**

The *A. baumannii* outbreak, was caused by MDR *A. baumannii*. The strains had widely spread among 12 departments especially in surgical intensive care unit (SICU), emergency intensive care unit (EICU) and the department of respiratory disease. The outbreak was more likely caused by the *A. baumannii* infected or carrier patients and EICU was its origin.

## 1. Background

The *Acinetobacter* species have recently attracted clinical and research interests, due to the increase of this genus being reported as a nosocomial pathogen. The *Acinetobacter* species are subclasses of the *Moraxellaceae* family which are strictly aerobic, Gram-negative, non-motile, non-lactose-fermenting, oxidase-negative, catalase-positive coccobacilli (1). So far, more than 30 genomic species have been identified in this genus, 17 of which have valid names (2). *Acinetobacter baumannii *is just one of many *Acinetobacter* species which can cause disease in humans, but in 2004, the centers for disease control (CDC) of USA reported that *A. baumannii* accounts for approximately 80% of all reported *Acinetobacter* infections. *A. baumannii* can be found in diverse sources such as foods, fresh water and soils. It is also a part of the indigenous flora of healthy human skin, especially at a low density and short-term duration (3).

*A. baumannii* is emerging as a cause of numerous global outbreaks which has demonstrated ever-increasing rates of resistances (4-7). *Acinetobacter* spp. are able to survive for a prolonged time on dry inanimate surfaces; the duration of which has been reported from one (8, 9) up to 5 months (10). *A. baumannii* strains are easily colonized in patients or medical equipment. Specific strains can attach to human epithelial cells through fimbriae or lipopolysaccharide side chains, bind to salivary mucins, or develop biofilm in contact with plastic or glass surfaces. The latter property is of particular clinical affiliation to the catheter-associated infections, (11) being resistance to the antimicrobial agents, and appearing as one of the most important factors of *Acinetobacter* infections in health-care settings.

## 2. Objectives

From April to June 2007, 53 patients had been infected with *A. baumannii* in emergency department and other units of PLA general hospital. We investigated the reason of outbreak, identified the source of the organism and found the suitable controlling methods.

## 3. Materials and Methods

### 3.1. Characterizing the Outbreak

PLA general hospital is a tertiary teaching hospital which offers acute medical and surgical services to thousands of people annually and admits about 80000 patients, 500 of which are accepted in the intensive care unit (ICU). In March 2007, it was found that the *A. baumannii* infections were very common in different wards of the hospital, while suddenly the number of infected cases increased in a short term with a similar drug resistance pattern were, mostly being drug and carbapenem multiple resistance.

### 3.2. Isolation and Identification of the Bacterial Strains

Written informed consent was obtained from each patient for bacterial isolation as well as clinical information usage. The study was approved by the ethnic review board of the PLA general hospital (ethnic number: 301-07-35).

The clinical *A. baumannii* strains were isolated between April and June 2007 from the respiratory care department, surgical intensive care unit (SICU), emergency intensive care unit and other wards. The environmental strains were collected simultaneously from the SICU regions (including the hands of medical staffs, patients’ skin, hospital beds, ventilator tubes and other equipment). The isolated strains were identified by Vitek assay (BiosMerieumx, France) in our microbiology laboratory using standard techniques. In order to guarantee 100% accuracy of the detected *A. baumannii* strains, the method of Ribotyping- “PCR amplification for BlaOXA-51-like carbapenemase gene” (12) was applied to identify all *A. baumannii* strains. Then isolated strains were stored at -70°C. At the meantime, the onset times were recorded according to patients’ case file.

### 3.3. Susceptibility Test

Minimal inhibitory concentration (MIC) assay was used to determine the drug sensitivity in all isolated strains by agar-dilution method and the corresponding range of drug concentrations were selected according to the recommendation of the national committee for clinical laboratory standards (2007 edition) (13). We used 1:9 ratios to prepare the drug agar panel. Cefoperazone- sulbactam MIC breakpoint was considered as the MIC breakpoint of cefoperazone; moxifloxacin MIC breakpoint was chosen according to the British society antimicrobial chemotherapy (BSAC) guidelines, (14) Tigecycline MIC breakpoint was used according to the FDA recommended value. In our study, isolated strains with intermediate susceptibility or resistance were defined as resistant.

### 3.4. Genetic Studies

Total genomic DNA was extracted from the isolated strains; DNAs were digested overnight by *ApaI* (Bio Link Inc., USA). A *Salmonella* serotype braenderup strain (H9812) was chosen as the universal size standard. Restriction fragments were separated on a 1% gel by pulsed-field gel electrophoresis (PFGE) using the CHEF Mapper® XA system (Bio-Rad Laboratories, USA) in 0.5 X TBE (Tris-borate-EDTA) buffer at 8°C. PFGE was performed with a 6.0 V/cm gradient, at 120° angle, and 7-20-s pulse times for 19 hours. Gel images were taken using the GEL DOC 2000 system (Bio-Rad Laboratories, USA) and analyzed by QUANTITY ONE software.

### 3.5. Analysis and Statistical Methods

The UPGMA (unweighted pair group method with arithmetic mean) method was applied for cluster analysis, using BioNumerics V4.6 software. The relationship between parenteral antibiotic using and carbapenem resistance rates in each unit was assessed by multiple linear regression analysis assays, using SPSS 17.0 software. For categorical variables, the Chi-squared test or Fisher’s exact test was used to compare the proportions. Statistical significance was considered as P ≤ 0.05.

## 4. Results

### 4.1. General Information

**Figure 1.**
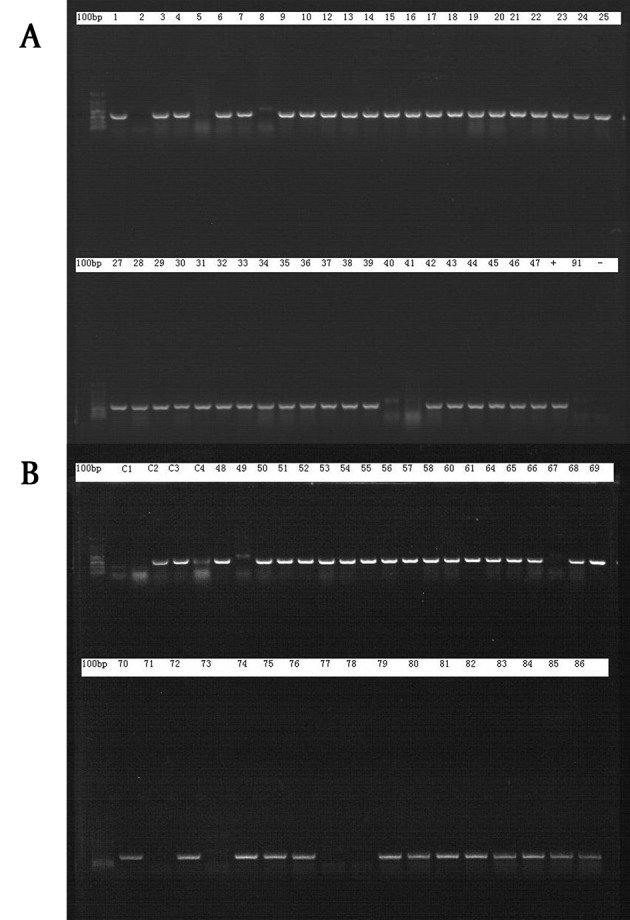
PCR Result of 86 Vitek Positive Strains, 72 of which were confirmed to be *A. baumannii* (A) Samples 1 - 47; (B) samples 48 - 86.

A total of 106 strains were successfully isolated, 86 of them showed positive results in Vitek assay, 72 of which were positive in PCR (Figure 1). After excluding the incomplete data, 69 strains of *A.baumannii,* isolated from 53 patients during the 3-month period, were successfully identified. Fifteen strains were collected from the respiratory care ward; among them, 14 were isolated from the surgical intensive care unit (SICU); 11 from the emergency intensive care unit; and 29 from the other wards. Another 28 environmental strains were collected simultaneously from the SICU regions (including the hands of medical staffs, patients’ skin, hospital beds, ventilator tubes and other equipment). Most of the patients were old (mean age: 56 ± 21 years) and the majority of them were male. Nineteen of them died and 34 were alive. The demographic data of these patients is shown in Table 1. 

**Table 1.** Demographic Data of 53 Patients From Whom *A. baumannii* Strains Were Isolated

Features	Variants
**Total patients, No.**	53
**Mean age, Mean **± **SD, y**	56 ± 21
**Male patients, No.**	33
**Patients in the ICU,No. **	40
**Death cases, No.**	19
**Average hospitalization, d **	74
**Cephalosporins users before confirmed, No.**	37
**β-lactam inhibitors users before confirmed, No.**	38
**Operative treatments, No.**	22
**Endotracheal intubations, No.**	35
**Catheter users, No.**	41
**Venous cathezeriation users, No.**	45
**Drainage tube users, No.**	22

### 4.2. Susceptibility Test

Table 2 shows the results of the susceptibility test. We found that Multi-drug resistance and Carbapenem resistant were the characteristics of the pathogen. The rate of resistance to Carbapenem was 75.4% (to meropenem was 85.5% and to Imipenem was 75.4%), Among the 19 tested agents, Colistin Sulphate and tigecycline showed high rates of antimicrobial activity against strains, The rate of susceptibility was 100% and 91.3% respectively. Moxifloxacin and minocycline had moderate antimicrobial activity against a baumannii, the rate of resistance being 87% and 36.2% respectively. Like the other agents, however, cefoperazone-sulbactam, piperacillin-tazobactam and Ticarcillin-clavulanic acid showed a low rate of antimicrobial activity, which the rate of resistance was 88.2%, 76.9% and 82.9% respectively.

**Table 2.** The MIC and Drug Resistance Rates of *A. baumannii* Strains to Antimicrobial Agents

Antibiotic	MIC^c^ Range (mg/mL)	MIC^50 ^^c^	MIC^90 ^^c^	Drug Resistance Rate
**Ampicillin**	16 - > 512	> 512	> 512	89.9
**Ampicillin-Sulbactam**	1 - 512	32	64	82.9
**Piperacillin-Tazobactam **	8 - > 512	256	512	76.9
**Ticarcillin-Clavulanic acid**	0.25 < - > 512	> 512	> 512	82.9
**Ceftazidime**	2 - > 512	128	256	91.3
**Cefepime**	1 - 256	64	64	80
**Cefotaxime**	8 - > 512	512	512	89.8
**Cefoperazone **	8 - >512	> 512	> 512	95.7
**Cefoperazone-Sulbactam**	2 – 256	64	64	88.2
**Imipenem**	0.125 - 128	32	64	75.4
**Meropenem**	0.25 - 128	32	64	85.5
**Polymyxin B sulfate**	0.5 - 2	1	1	0
**Gentamicin**	0.5 - >256	> 256	> 256	87
**Amikacin**	2 - >512	> 512	> 512	84
**Minocycline**	0.25 – 32	8	32	36.2
**Tigecycline ** ^**a**^	0.125 - 8	1	2	8.7
**Levofloxacin**	0.125 - 64	8	32	84
**Moxifloxacin ** ^**b**^	0.06 - 64	8	32	87
**Trimethoprim-Mulphamethoxazole**	0.025- > 32	> 32	> 32	85.5

^a^ Tigecycline was referenced according to the CLSI *Enterobacteriaceae* information.

^b^ Empirical clinical drug usage, referenced break point of BSAC (British society antimicrobial chemotherapy).

^c^ MIC, Minimum inhibitory concentration; MIC^50^, Minimum Inhibitory Concentration required to inhibit the growth of 50% of organisms; MIC ^90^, Minimum Inhibitory Concentration required to inhibit the growth of 90% of organisms

### 4.3. Genetic Studies

**Figure 2.**
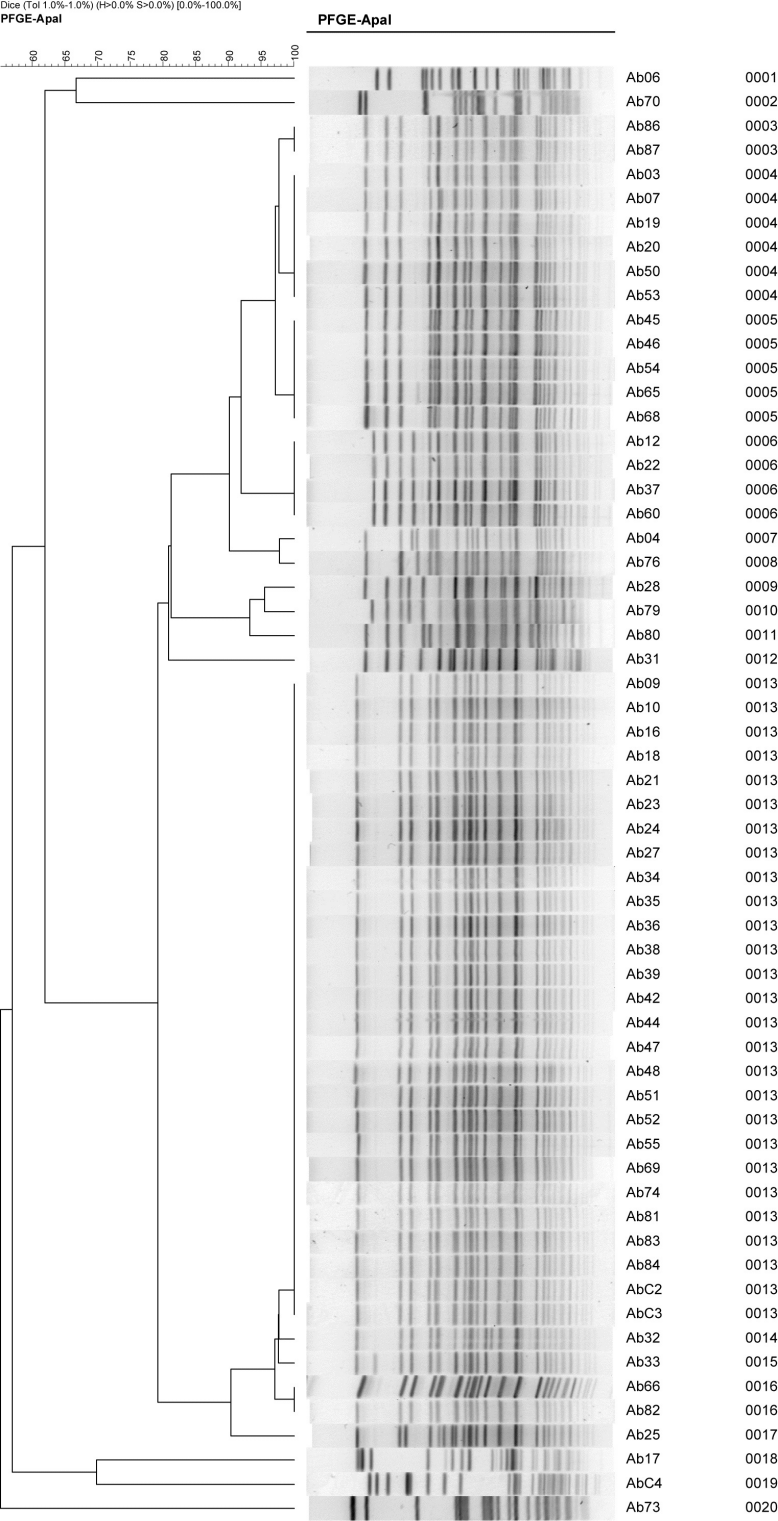
The PFGE Clustering Analysis Results of 62 *A. baumannii *Strains

The PFGE result (Figure 2) revealed that there were nine unique clones and the most prevalent types were A, B and C. Type A and C have three subtypes, type B has four subtypes and type D to I are individual. The environmental concerned strains are not relevant to the epidemic strains A, B and C. Combined with the in vitro antimicrobial susceptibility test results, we concluded that all type A strains were CRAb, while only a part of type B and C strains were CRAb.

Carbapenem, imipenem and meropenem showed no sensitivity differences to MDR *A.baumannii* at this outbreak; the moxifloxacin resistance was significantly different in type C strains, compared with type A and B. We chronologically and separately investigated the three clone types (A, B and C), and found out that the type A and C clones emerged in the emergency department earlier.

## 5. Discussion

*A. baumannii* is widely spread in the nature, which could be collected from soil, water, (15) humidifiers, pillows, ventilators, medical staff skins and surface of other items (16-18). Its survival at the surfaces has made it the hospital infection pathogen (19). In our study, the frequency of the collected positive *A. baumannii* samples was 21.7% in the respiratory care medicine department, 20.3% in the intensive care unit and 15.9% in the emergency care unit. Generally, mechanical ventilation is often considered as the most important *A. baumannii* pneumonia-causing factor (19-23). Application of antibiotics in patients with severe infections increases the opportunity of antimicrobial selective pressure accordingly (24). Correspondingly, the number of infections caused by multi-drug resistant *A. baumannii* strains are increasing. Current reports showed that only a minority of antibacterial drugs such as polymyxin, tigecycline and formulations containing Sulbactam can be used for the treatment of *A. baumannii (*25-27*).*

In this study, the isolated strains had similar resistance characteristics, the CRAb rate was 75.4%, making it the main cause of the *A. baumannii *outbreak. The PFGE analysis resultrevealed the preliminary answer of the outbreak: MDR *A. baumannii* occurred in 12 medical rooms of seven wards located in three medical buildings, the A1 type of *A. baumannii* was isolated from the hands of nursing staffs as well as the patients’ body fluids; meanwhile, isolated samples from nurses’ washed hands were negative. These results demonstrate that the CRAb on the hands of care staffs was the important source of pathogenic *Acinetobacter* infections in the hospital. Secondly, we analyzed the transmission pattern of MDR *A. baumannii* in three major departments-the respiratory care, emergency and the SICU department, which showed that most of the patients in the respiratory care department were only staying in that specific ward, not having much contact with others; Emergency department had complex patients and diseases with the most transferring frequency, patients in SICU generally had serious conditions and more transferring thus the transfer of patients possessing MDR *A. baumannii* infection was the main reason of the spread between the units.

Accordingly, we investigated the patients with MDR *A. baumannii* infection carefully and found the main popular type in the respiratory care ward, which was type A and not C. Only one isolated B2 type (No. 68) was transferred from the SICU. In the SICU, A and B types were the epidemic strains and only one type C specimen (No. 79) was confirmed to be transferred from the emergency ICU. Emergency department had all types A, B and C epidemic strains. According to the medical records analysis results, emergency rooms have the highest number of epidemic strains, thus we deduced the possible pattern of MDR *A. baumannii* transmission outbreak in the hospital: emergency department has significant possibility as the pathogen occurrence location, due to the high number of patients' transfers in the emergency and SICU departments compared with the others.

Patients with a clinical *A. baumannii* infection have to pay additional charges because they have to stay in the hospital for an average of 13 days longer than a patient without *A. baumannii* infection (28). *A. baumannii* may not be particularly virulent, but it can cause unnecessary diseases and impose extra expenses on critically ill patients, thus the transmission of such pathogen should be limited.

In conclusion, *A. baumannii* infections are associated with considerable morbidity and mortality. Therefore, proceedings such as strengthening the concept of hand washing in clinical staffs as well as strengthening the isolation of patients with MDRAb infection, and effective anti-infection treatments for patients could effectively prevent the spread of *A. baumannii*. 
